# Alterations in gut microbiota of abdominal aortic aneurysm mice

**DOI:** 10.1186/s12872-020-01334-2

**Published:** 2020-01-28

**Authors:** Jiahe Xie, Weiling Lu, Lintao Zhong, Yuhua Hu, Qingrui Li, Rongming Ding, Zhonggao Zhong, Ziyou Liu, Hai Xiao, Dongming Xie, Guofu Zheng, Bo Ye, Yiming Zhong, Zuheng Liu

**Affiliations:** 1 Department of Cardiology, First Affiliated Hospital of Gannan Medical University, Ganzhou, China; 2 State Key Laboratory of Organ Failure Research, Department of Cardiology, Nanfang Hospital, Southern Medical University, Guangzhou, China; 3 Key Laboratory of Prevention and Treatment of Cardiovascular and Cerebrovascular Diseases, Ministry of Education, Gannan Medical University, Ganzhou, People’s Republic of China; 4 Department of Cardiology, Zhuhai Hospital, Jinan University, Zhuhai, China; 5 Department of Vascular Surgery, the Affiliated Ganzhou Hospital of Nanchang University, Guangzhou, China; 6 Key Laboratory for Organ Failure Research, Ministry of Education of the People’s Republic of China, Southern Medical University, Guangzhou, China

**Keywords:** Abdominal aortic aneurysm, Gut microbiota, *Akkermansia*

## Abstract

**Background:**

The gut microbiome plays an important role in various cardiovascular diseases, such as atherosclerosis and hypertension, which are associated with abdominal aortic aneurysms (AAAs).

**Methods:**

Here, we used 16S rRNA sequencing to explore gut microbiota in C57BL ApoE^−/−^ mice with AAAs. A mouse model of abdominal aortic aneurysms was induced with angiotensin II (Ang II) (1000 ng/min per kg). On day 28 after the operation, fecal samples were collected and stored at − 80 °C until DNA extraction. We determined the relative abundances of bacterial taxonomic groups using 16S rRNA amplicon metabarcoding, and sequences were analyzed using a combination of mother software and UPARSE.

**Results:**

We found that the gut microbiome was different between control and AAA mice. The results of correlation analysis between AAA diameter and the gut microbiome as well as LEfSe of the genera Akkermansia, Odoribacter, Helicobacter and Ruminococcus might be important in the progression of AAAs.

**Conclusions:**

AAA mice is subjected to gut microbial dysbiosis, and gut microbiota might be a potential target for further investigation.

## Background

Abdominal aortic aneurysm (AAA), a vascular disease with high disability and mortality, is associated with atherosclerosis and hypertension [1]. An abnormal vascular inflammatory response and abnormal vascular structure trigger the onset of aortic aneurysms.

Recent studies have suggested that gut microbiota probably participate in host inflammation and the formation of atherosclerosis and hypertension [2, 3]. Gut microbiota have been shown to be closely related to systemic inflammation by generating some toxic metabolic factors or by releasing lipopolysaccharides [4]. Any factors that influence the intestinal microenvironment likely interrupt the balance in gut microbiota, ultimately leading to changes in the gut microbiome. For example, high choline intake elevates the level of blood trimethylamine oxide (TMAO), a metabolic factor that promotes cordial hypertrophy and ultimately increases the incidence of various cardiovascular- and cerebrovascular-related diseases [5–9], while even changes in diet can change the gut miceobiome [10–12]. All these factors can change the gut microbiome.

Therefore, focusing on the gut microbiome might provide some possible mechanism of AAAs. In this study, we applied Ang II-induced AAA to ApoE^−/−^ mice and investigated alterations in the gut microbiome.

## Methods

### Abdominal aortic aneurysm mouse model

Five- to six-month-old male C57BL ApoE^−/−^ mice were obtained from the laboratory of the animal center of Gannan Medical University and were used in this study. All mice were housed in cages and bred in a temperature-controlled room maintained at 22–26 °C on a 12-h light-dark cycle with standard food and water. This study was approved by the Gannan Medical University animal ethics committee, and the use of animals in this study was in compliance with the Guide for the Care and Use of Laboratory Animals (NIH, 8th Edition, 2011). The mice used in this study were anesthetized with a mixture of xylazine (5 mg/kg) and ketamine (100 mg/kg) by intraperitoneal injection. A mouse model of abdominal aortic aneurysm or sham was induced with angiotensin II (1000 ng/min per kg, *n* = 17) or saline (*n* = 8) by Alzet Osmotic Pump implantation, which has been described previously [13]. After 28 days, fecal samples were collected and stored at − 80 °C until DNA extraction. After fecal samples collection, mice were sacrificed by overdose of pentobarbital (500 mg/kg), then the abdominal aorta were separated. Among them, four mice died in observation period which probably caused by the rupture of arterial aneurysm, while another 3 mice were excluded because the increased vessel diameter were less than 50%.

### DNA extraction

Samples were stored at − 80 °C until DNA extraction. The DNA was extracted from 200 mg samples using the QIAamp DNA Stool Mini Kit (QIAGEN, Hilden, Germany) following the manufacturer’s instructions. The DNA concentration and purity were checked by running the samples on 1.0% agarose gels.

### 16S rRNA amplification and sequencing

The methods were described previously [14]. Briefly, 16S rRNA genes were amplified by using V3-V4 regions bacterial primers (357F 5′- ACTCCTACGGRAGGCAGCAG-3′ and 806R5’- GGACTACHVGGGTWTCTAAT − 3′). The primers also contained the Illumina 5′ overhang adapter sequences. The libraries were sequenced on the MiSeq PE300 sequencing platform using a MiSeq v3 Reagent Kit (Illumina).

### Bioinformatic analysis

Bioinformatic analysis were described in previous study [15]. Briefly, after the raw data were demultiplexed based on the barcode, low-quality base pairs were removed by using parameters with SLIDINGWINDOW: 50:20 MINLEN: 50. Then, 16S rRNA sequences were analyzed by using mothur software (version 1.33.3), UPARSE (usearch version v8.1.1756,), and R (version 3.2.3). For the alpha-diversity analysis, Shannon, Chao1, ACE and PD_whole_ tree were calculated by using mother, while the beta-diversity metrics, the weighted and unweighted UniFrac distance were calculated by using mothur and visualized with principal coordinate analysis (PCoA) and tree by R.

### Statistical analysis

Quantitative data were displayed as mean ± SEM (standard error of mean). Comparison between two experimental groups was based on a two-tailed t-test. In all analyses, differences were considered statistically significant at a value of *P* < 0.05.

## Results

### The abdominal aortic aneurysm model was induced by angiotensin II

**Fig. 1:**
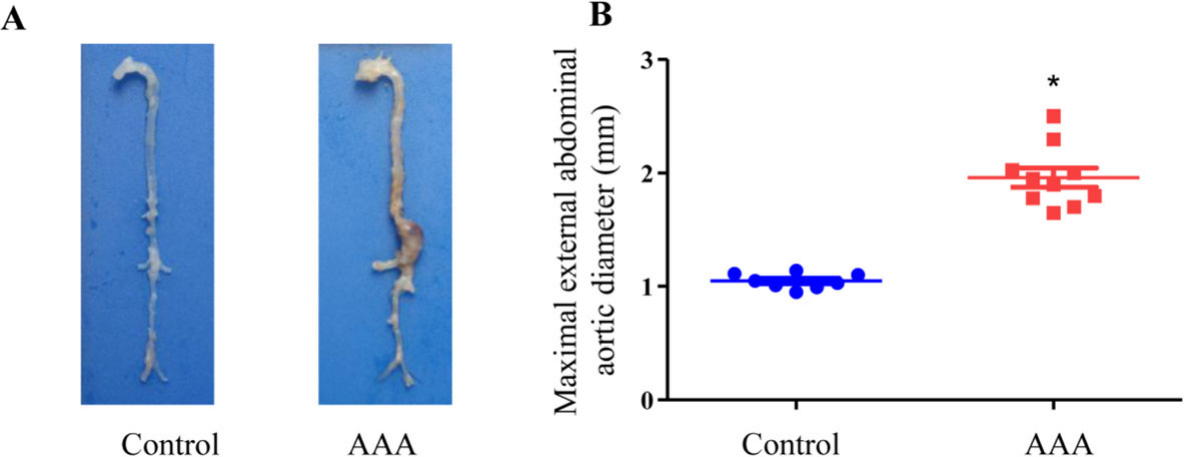
**a** Representative images of abdominal aortic aneurysms. **b** A scatter plot of maximal external abdominal aortic diameter. *P < 0.05, *n* = 8–10

After an overdose of anesthesia, the mice were euthanized. Then, the abdominal aortas were dissected, and the AAAs were visualized, as shown in Fig. 1a. The maximal external abdominal aortic diameter was measured using ImageJ software, which showed a significant difference between the AAA and control groups (*n* = 8–10, *P* < 0.05).

### The gut microbial composition changed in the abdominal aortic aneurysm mouse model

**Fig. 2:**
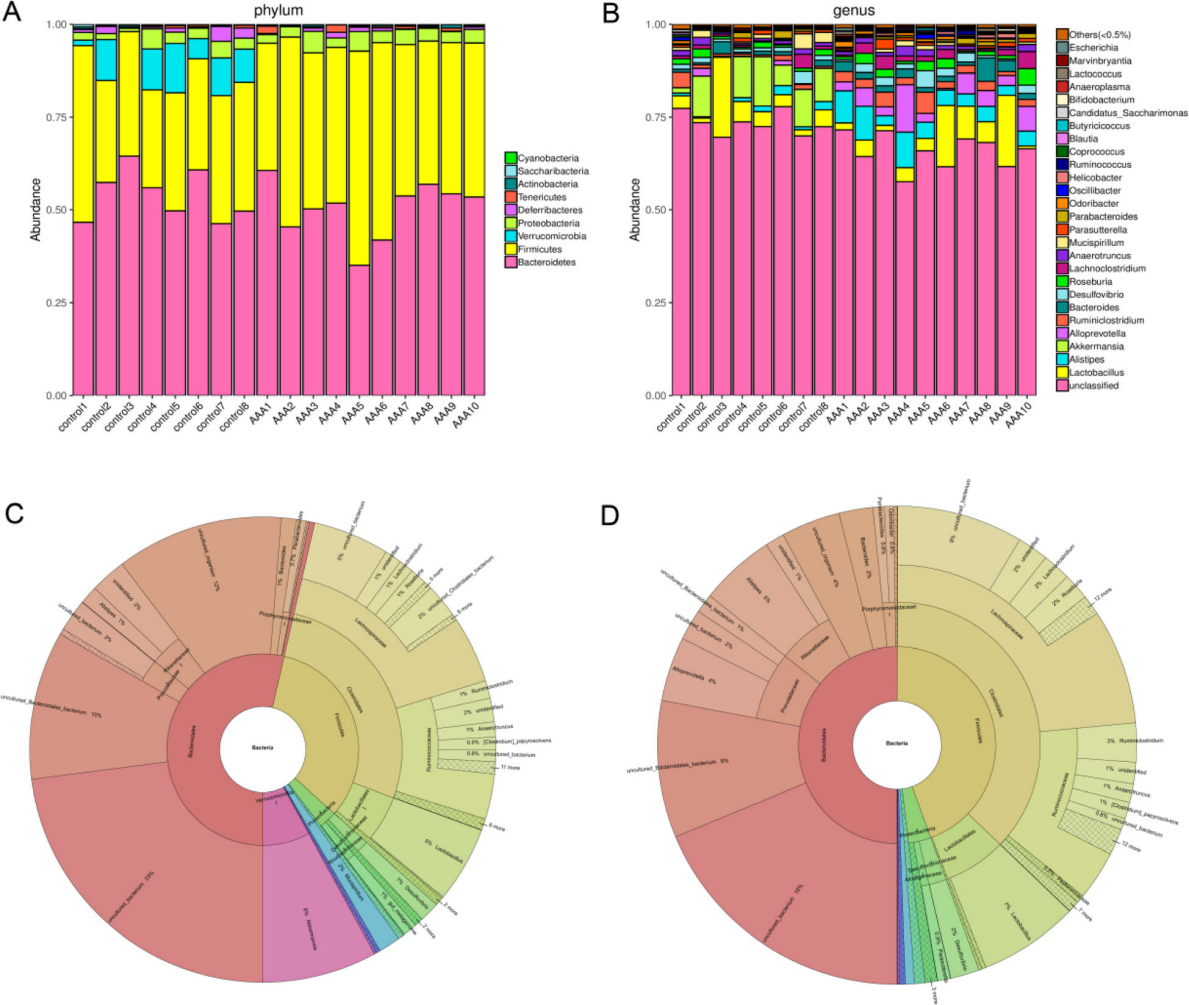
Relative abundance is shown at the **a** phylum and **b** genus levels. **c** - **d** Krona analysis of the bacterial community structure between the control and AAA groups. The composition is based on 16S rRNA sequencing

To explore the gut microbiome in AAA mice, fecal samples were collected before the mice were sacrificed. After bioinformatic analysis, we found that Bacteroidetes, Firmicutes, Verrucomicrobia and Proteobacteria in the taxonomic composition plots at the phylum level represented most of the bacterial community of the gut (Fig. 2a). At the genus level, we found that *Akkermansia* was reduced in AAA mice (Fig. 2b), and this result was also demonstrated by Krona analysis (Fig. 2c-d).

### The alpha and beta diversities of the gut microbiome between the AAA and control groups

**Fig. 3:**
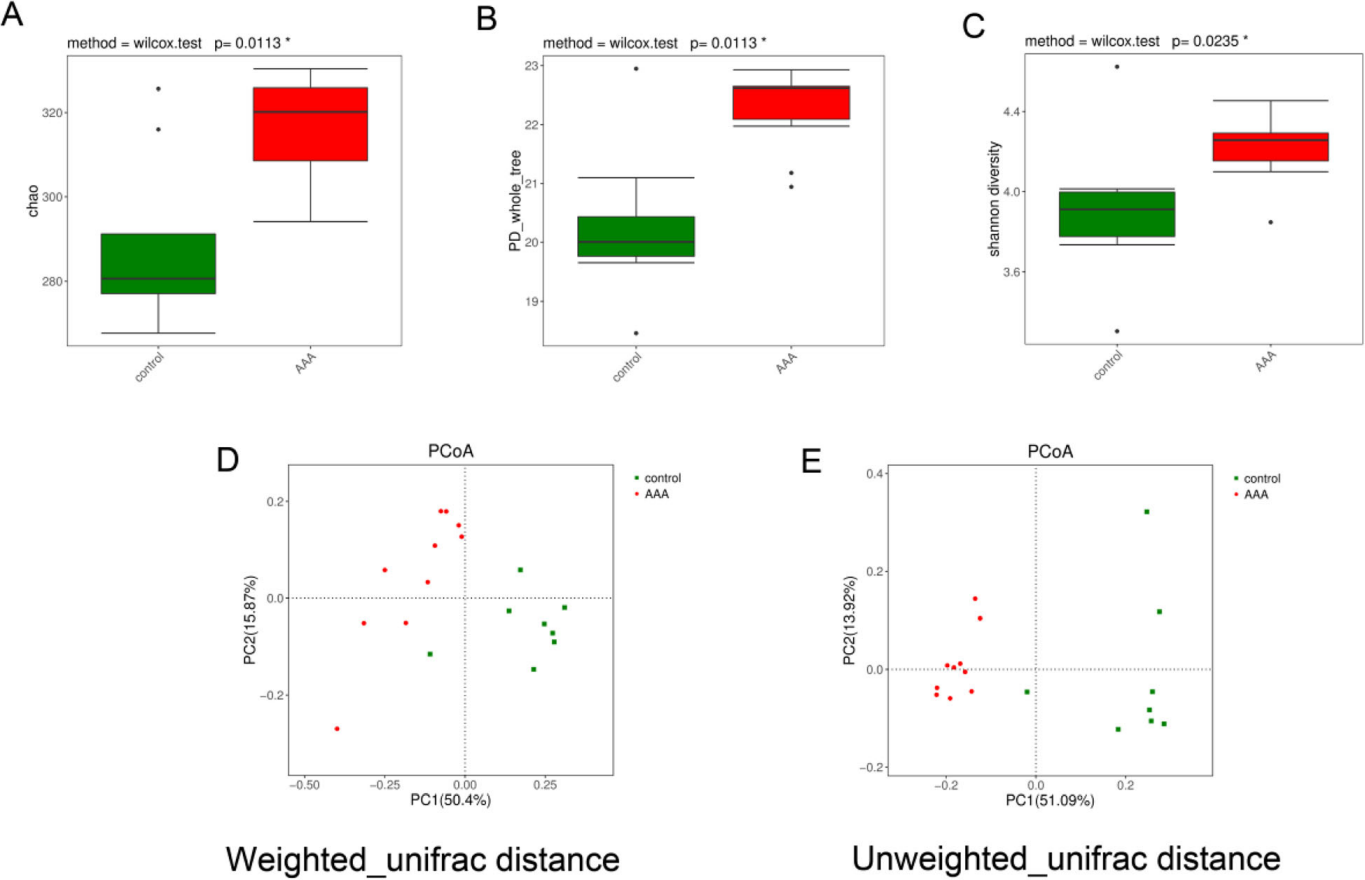
Alpha diversity of the **a** Chao index, **b** PD_whole tree and **c** Shannon index between the control and AAA groups. Principal coordinate analysis (PCoA) of gut microbiota for **d** Weighted_unifrac distance and **e** Unweighted_unifrac distance from AAA mice and control mice. (Red dots, AAA group; Green dots, control group)

Then, principal coordinate analysis (PCoA) of gut microbiota from AAA mice and control mice were performed. We found that AAA mice had higher (Fig. 3a) Chao index (*P* < 0.05), (Fig. 3b) PD_whole tree (P < 0.05) and (Fig. 3c) Shannon index (*P* < 0.05) levels than control mice. In addition, beta diversity was shown by (Fig. 3d) Weighted_unifrac distance (*P* < 0.05) and (Fig. 3e) Unweihted_unifrac distance (*P* < 0.05). Together, these results indicate that the gut microbiome might participate in the progression of AAA.

### The LDA effect size analysis (LEfSe) revealed the differences between the AAA and control groups

**Fig. 4:**
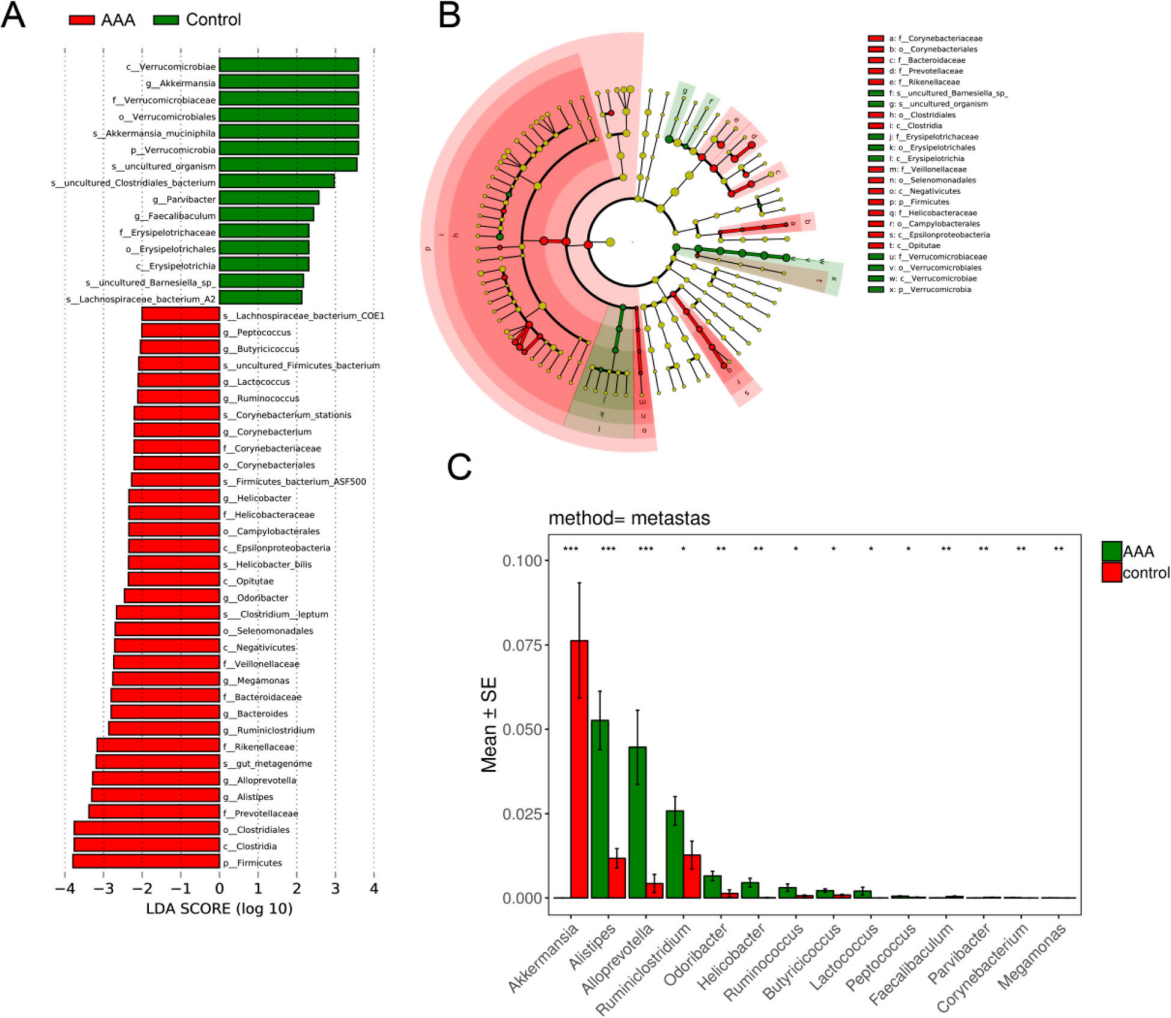
**a** – **b** Linear discriminant effect size analysis (LEfSe) between the control and AAA mice. **c** The relative abundance at the genus level between the AAA and control groups. *P < 0.05, **P < 0.01, ***P < 0.001

To identify distinctive features between these groups, LEfSe was performed (Fig. 4a-b). The results of an LDA effect size analysis showed that species from *AAkkermansia. muciniphila* and *Lachnospiraceae bacterium A2* were significantly higher in the control group than in the AAA group, while six species were increased in AAA mice: *Lachnospiraceae bacterium, COE1*, *Corynebacterium stationis, Firmicutes Bacterium ASF500, Helicobacter bilis* and *Clostridium leptum* (Fig. 4a-b)*.* Differential analysis by metastas also revealed a similar result (Fig. 4c); thus, *Akkermansia, Alistipes, Alloprevotella, Ruminiclostridium, Odoribacter* and *Helicobacter* might be important in AAAs.

### The correlation between the abdominal aortic diameter and the gut microbiome

**Fig. 5:**
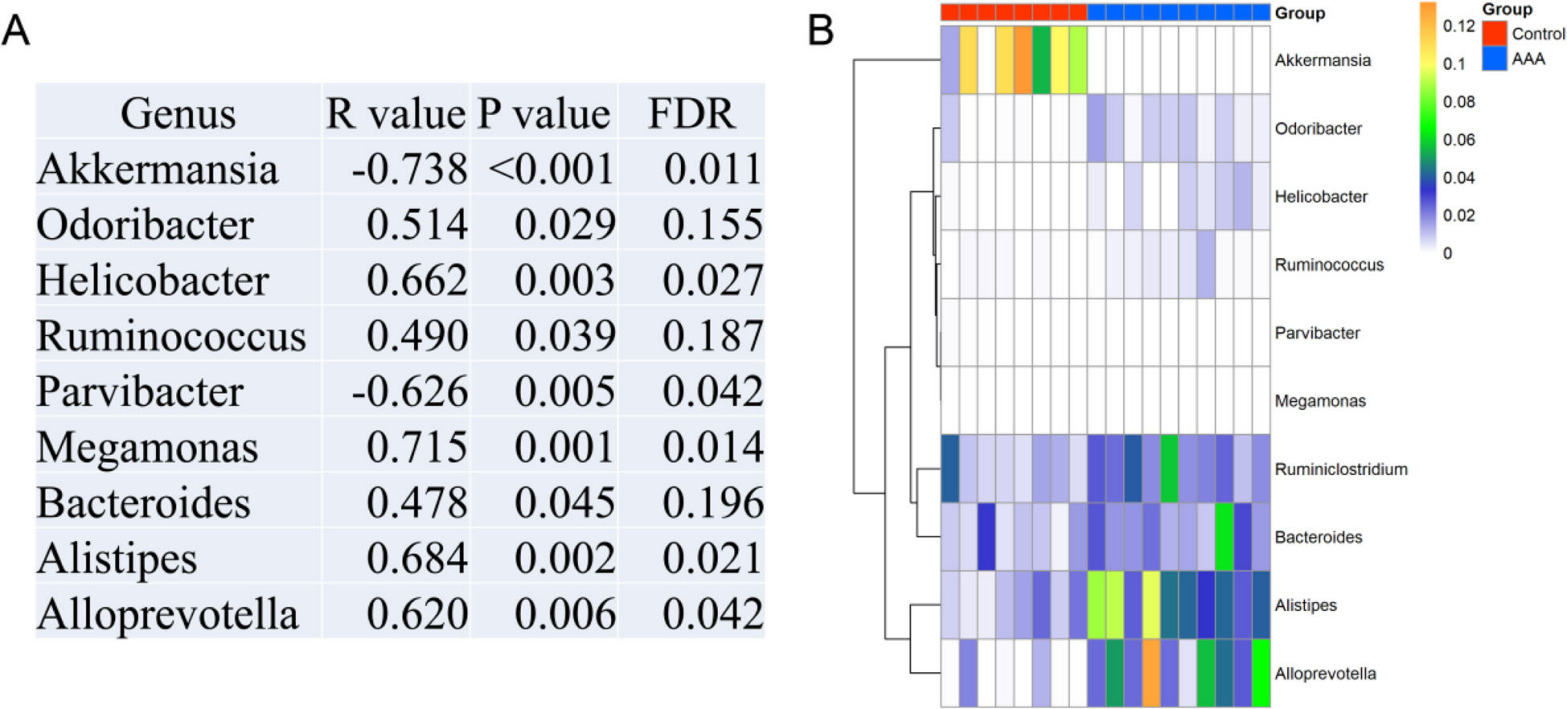
**a** Correlations between the gut microbiome at the genus level and the maximal external abdominal aortic diameter. **b** A heatmap of the gut microbiome between the control and AAA groups

To explore the correlation between the severity of AAA and the gut microbiome, Spearman correlation was used. The R value, *P* value and FDR are shown in Fig. 5a, while the heat map is shown in Fig. 5b. We found that 2 genera (*Akkermansia* and *Parvibacter*) were negatively correlated with the diameter of AAA, whereas 7 genera (*Odoribacter*, *Helicobacter*, *Ruminococcus*, *Megamonas*, *Bacteroides*, *Alistipes*, and *Alloprevotella*) were significantly positively correlated with the diameter. These findings suggest that *Akkermansia* and *Parvibacter* might be essential in the treatment of AAAs.

## Discussion

In the present study, we found that abdominal aortic aneurysm (AAA) mice were subject to gut microbial dysbiosis. Although gut microbiome alterations emerged after the induction of AAAs, gut microbial dysbiosis likely inversely promoted the course of disease. Thus, exploring the gut microbiome in AAAs might provide some clues in the progress of AAAs.

First, we found that the gut microbial community was different between the AAA group and the corresponding control group. This result might be due to the challenge of angiotensin II, an endogenous, rigorous vasoconstrictor that can promote cardiac hypertrophy and impair vascular endothelial cells [16]. The stimulation of Ang II might lead to vasoconstriction in the intestine and thus further interrupt the gut microbiome [17]. Similar results were found in high blood pressure patients, as we found that the genus *Alistipes*, harbored in HBP patients [17], was positively correlated with the diameter of an AAA. However, our findings in the gut microbiome were not completely consistent with previous findings in a hypertension mouse model induced by Ang II [17]. These differences might be due to the knock-out of the *ApoE* gene, as gut microbiota interact with host genes [18]. Another explanation is the different symbiotic bacteria in these studies, as different regions also influence the gut microbiome [19].

We also found that *A. muciniphila,* a newly identified probiotic reported in recent years [20], was significantly reduced in the AAA mice. It is not surprising that *A. muciniphila* might be involved in the progression of AAAs because it exerts beneficial effects in various diseases including atherosclerosis [21] and dyslipidemia [22]. Other studies also found that *Akkermansia* decreased with increasing age in Tibetan minipigs [23], and it was found to be induced by some types of traditional Chinese medicine [24].

*A. muciniphila* is a kind of mucin-producing bacteria that can repair damage to the intestinal barrier in atherosclerosis models of ApoE^−/−^ mice [21]. In addition, we previously found that exercise training could increase the abundance of *Akkermansia* [20]*.* These results indicate that *A. muciniphila* is likely essential in the host body and that it might be essential in the progress of AAAs.

In addition, a species belonging to *Helicobacter* was also increased in AAA mice. This result was similar to that of our previous study in myocardial infarction mice, as *Helicobacter* was negatively associated with left ventricular ejection fraction in MI mice [20], indicating that *Helicobacter* might be a common pathogen underlying some cardiovascular diseases.

The AAA model was established with a sustained infusion of angiotension II. Although previous studies have shown that the gut microbiome changes after challenge with angiotensin II or dyslipidemia, they have not investigated both of these effects. Additionally, AAA patients in the clinic always exhibit dyslipidemia and abnormal blood pressure. Nevertheless, further studies are still required to verify the function of these diseases.

There are still some limitations should be concerned. First, the mechanism of AAA seems more complicated in clinical practice rather than the abnormal of renin-angiotensin system or simply the deficiency of *ApoE* gene. Second, the reduction of *A. muciniphila* in AAA mice is an observational result, more data form human analysis is needed, and the underlying mechanisms of *A. muciniphila* should be investigated in the future.

## Conclusions

AAA mice is accompanied with gut microbial dysbiosis.

## Data Availability

All relevant data is presented in the manuscript and the datasets used and analyzed during the current study are available from the corresponding author on reasonable request. The sequences were deposited in the European Nucleotide Archive (ENA) under the accession number PRJEB34405.

## Abbreviations

AAAs: Abdominal aortic aneurysms
Ang II: Angiotensin II
LEfSe: LDA effect size analysis
PCoA: The principal coordinate analysis
TMAO: Trimethylamine oxide
